# From Resistance to Redesign—The Emerging Logic of Hybrid Care in Treatment-Resistant Depression

**DOI:** 10.3390/brainsci16060612

**Published:** 2026-06-04

**Authors:** Federico Mucci, Riccardo Gurrieri, Siham Bouanani, Matteo Gambini, Gerardo Russomanno, Donatella Marazziti

**Affiliations:** 1 Department of Psychiatry, Lucca Zone, Azienda USL Toscana Nord Ovest, 55100 Lucca, Italy; 2 Health Science Interdisciplinary Center, Sant’Anna School of Advanced Studies, 56127 Pisa, Italy; 3 Department of Clinical and Experimental Medicine, Section of Psychiatry, University of Pisa, 56126 Pisa, Italygambinimatteo1996@gmail.com (M.G.);

**Keywords:** treatment-resistant depression, TRD, major depressive disorder, novel approaches, depression biomarkers

## Abstract

**Highlights:**

**What are the main findings?**

- The TRD trial landscape is dominated by device-based neuromodulation (48.1%) and pharmacological strategies with novel mechanisms (36.3%); biologic/novel agents (6.8%) and digital–hybrid programs (2.1%) form smaller strata.
- Trials are predominantly mid-phase with small-to-moderate sample sizes and heterogeneous endpoints, although 63.3% adopt standard clinician-rated scales.

**What are the implications of the main findings?**

- The TRD trial ecosystem is structured around two co-active developmental tracks—somatic neuromodulation and novel-mechanism pharmacology—with biologic/novel agents emerging and digital programs still marginal.
- Greater harmonization of clinician-rated endpoints with cognitive and functional measures, longer follow-up, and biomarker-informed stratification are needed.

**Abstract:**

**Background/Objectives:** Treatment-resistant depression (TRD) remains one of the most urgent unmet needs in psychiatry, while its therapeutic pipeline is evolving rapidly. To characterize current development trajectories, we conducted a registry-anchored mapping of interventional trials in adults with major depressive disorder and treatment resistance (MDD-TRD), with the aim of defining the distribution of intervention types, endpoint choices, and key design features across the active trial landscape. **Methods:** We systematically searched ClinicalTrials.gov, the EU Clinical Trials Information System, and ISRCTN for interventional MDD-TRD trials registered up to 18 September 2025. After data cleaning and cross-registry deduplication, 237 unique trials were retained. Interventions were categorized through a taxonomy distinguishing device-based neuromodulation, pharmacological strategies, biologic/novel agents, multimodal non-digital combinations, digital–hybrid programs, psychotherapy, and lifestyle interventions, with classification anchored on structured registry intervention tags and whole-word matching across title and intervention text. Primary endpoints were flagged as standard when they explicitly referenced the Montgomery–Åsberg Depression Rating Scale or Hamilton Depression Rating Scale. We also examined developmental phase, sample size, and recurrent methodological features. **Results:** Device-based neuromodulation accounted for the largest share of the active pipeline (114/237, 48.1%), followed by pharmacological strategies (86/237, 36.3%), biologic/novel agents (16/237, 6.8%), and multimodal non-digital combinations (11/237, 4.6%). Digital–hybrid programs represented a small but distinctive stratum (5/237, 2.1%), with the remaining records comprising lifestyle interventions (3/237, 1.3%) and psychotherapy (2/237, 0.8%). Standard clinician-rated primary endpoints were used in 63.3% of studies. Trial development was concentrated in mid-phase designs, whereas sample sizes were generally modest (median 49; interquartile range, 19–87). Across modalities, increasing attention was directed to durability of response, functioning, and patient-reported outcomes, with adaptive and enrichment-based designs appearing with greater frequency. **Conclusions:** The contemporary TRD trial ecosystem is structured around two co-active developmental tracks—device-based neuromodulation and pharmacology with novel mechanisms—accompanied by a smaller but measurably expanding biologic/novel stratum and a still-marginal digital–hybrid presence. This registry-based mapping provides a near-real-time overview of the field and may support future harmonization of trial endpoints and design standards.

## 1. Introduction

Major depressive disorder (MDD) is one of the leading causes of disability worldwide, and is associated with deep personal suffering, excess mortality, and staggering economic consequences. According to the World Health Organization, MDD represents the single largest contributor to loss of healthy life years, a burden that the COVID-19 pandemic has further amplified. Although conventional antidepressants, structured psychotherapies, and neuromodulation techniques have demonstrated efficacy, a substantial proportion of patients fail to achieve satisfactory outcomes, even after multiple sequential interventions, thus entering the clinical trajectory commonly described as treatment-resistant depression (TRD) [1].

Despite decades of research, a universally accepted definition of TRD with predictive validity is still lacking. Operationalizations vary considerably, ranging from failure of two adequate antidepressant trials to more complex multidimensional criteria incorporating duration of episode, comorbidity, and patient-reported outcomes. This definition heterogeneity translates into widely divergent prevalence estimates, yet epidemiological extrapolations suggest that more than 100 million people worldwide may currently fulfil at least one definition of TRD [1,2]. Such figures underscore the paramount degree of the problem while highlighting it as a pressing public health priority. Within this landscape, intranasal esketamine became the first pharmacological agent to be formally approved with a specific indication for TRD by both the FDA and the EMA, joining established somatic strategies—most prominently ECT and non-convulsive neuromodulation—among the therapeutic options currently available to clinicians. Yet, the persistence of substantial non-response rates even after these interventions [2,3] continues to motivate the search for new mechanisms of action, delivery formats, and stratification strategies.

Treatment-resistant depression (TRD) continues to be defined within a shifting methodological framework in which competing operational criteria, heterogeneous staging systems, and variable thresholds for what constitutes an “adequate” treatment failure do not merely reflect academic disagreement but translate into the enrollment of profoundly different patient populations across studies, with consequences that reverberate through effect sizes, guideline recommendations, reimbursement policies, and, ultimately, the quality of care delivered [1,3]. In this setting, prospectively charting ongoing interventions through clinical-trial registries provides a complementary vantage point to the published record, allowing observation—near real-time—of where investment is directed, how mechanisms of action are distributed, and how programs mature across phases [1,2,3]. In this sense, a registry-based mapping of ongoing trials—classifying interventions according to mechanism, design, endpoints, and enrichment strategies—becomes essential to determine whether the field is moving toward genuinely stratified, biomarker-informed care or merely multiplying undifferentiated therapeutic options [2,3]. In a previous narrative review focused primarily on the potential role of botulinum toxin in depressive disorders, we briefly discussed the investigational pipeline for TRD as part of a broader argument, observing in qualitative terms that ongoing clinical trials provide a vantage point complementary to the published literature on where research efforts are currently directed [4]. That excursus did not aim at a systematic registry-anchored mapping; the present work is the systematic exercise that the earlier observation anticipated, with explicit eligibility criteria, deduplication across three registries, a pre-specified taxonomy, and a transparent operationalization of treatment resistance. That excursus suggested how the study of ongoing clinical trials might offer a different vantage point from that provided by the published literature, not as a substitute but as a complementary source of information on where research efforts are currently directed and how new strategies are being tested.

The present work aims at further developing that preliminary observation into a more systematic exercise by interrogating trial registries and organizing the available data in order to sketch a preliminary map of the therapeutic approaches under evaluation. Such a map cannot provide certainties, but it may help to frame the directions in which the field is moving, the relative weight of competing mechanisms, and the methodological gaps that remain to be addressed before these innovations can meaningfully alter the clinical course of TRD.

The present scoping review addresses three explicit questions: how active interventional programmes for adult major depressive disorder meeting treatment-resistance criteria are distributed across mechanism-of-action families; how they cluster across development phases and primary-endpoint choices; and how key design features—sample size, recruitment status, and sponsorship—characterize the contemporary pipeline. In Population–Concept–Context (PCC) terms, the Population is adult MDD with treatment resistance, the Concept is the interventional developmental pipeline (intervention classes, phases, endpoints, design features), and the Context is the set of three international clinical-trial registries—ClinicalTrials.gov, the EU Clinical Trials Information System, and ISRCTN—interrogated with a common cut-off date of 18 September 2025. Within this design, the active pipeline is organized along seven analytic categories—device-based neuromodulation, pharmacological strategies distinct from ketamine and esketamine, biologic and novel agents, multimodal non-digital combinations, digital–hybrid programs, psychotherapy, and lifestyle interventions—with attention to how each family is positioned across developmental phases, endpoint choices, and sample-size profiles.

Further elaboration on the epidemiologic burden, treatment paradigms, and definitional frameworks of TRD is provided in the Supplementary Materials.

## 2. Materials and Methods

**Figure 1:**
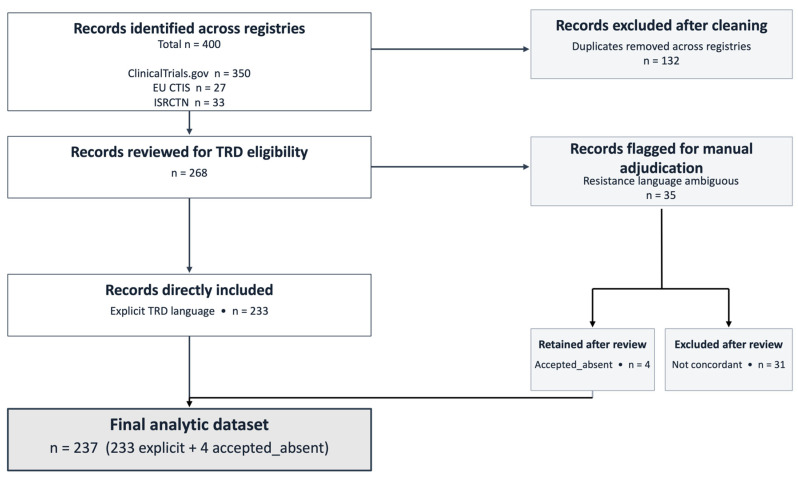
Records identified across ClinicalTrials.gov, the EU Clinical Trials Information System (EU CTIS), and ISRCTN at the 18 September 2025 cut-off. Per-registry counts sum to 410; the difference relative to the unique total (*n* = 400) reflects multi-registered trials detected at the cleaning and deduplication stage and consolidated into the corresponding exclusion node. Arrows indicate the flow of record screening and selection.

This scoping review was designed and reported following the PRISMA extension for Scoping Reviews (PRISMA-ScR). The completed PRISMA-ScR checklist and the flow diagram (Figure 1) are provided with this manuscript.

This review was conceived as a registry-anchored, interventional pipeline mapping for adult major depressive disorder with treatment resistance (MDD TRD), in continuity with a previous scoping of the topic embedded within a broader appraisal of botulinum toxin in depression [4]. Consistent with the persistent heterogeneity of TRD definitions across the literature [1,2,3], we chose to adopt an approach that privileges verbatim operationalizations as recorded in trial registries, while imposing only a minimal and transparent harmonization layer to safeguard reproducibility. A detailed description of the registry workflow, including the filtering and deduplication hierarchy, is summarized in Figure 1. Full details of the registry queries, tagging procedures, endpoint definitions, and cleaning rules are available in the Supplementary Information (see Supplementary Methods).

### 2.1. Data Sources and Search Strategy

We interrogated three primary registries: ClinicalTrials.gov (United States), the EU Clinical Trials Information System (CTIS; European Union/EEA), and the ISRCTN Registry (United Kingdom). Because these platforms do not share a controlled vocabulary and differ in the granularity of their structured fields, searches were tailored to each environment but anchored to a single conceptual core. The registry snapshot was locked on 18 September 2025; any amendments or post hoc updates after this date were not considered in the analytic dataset. No review protocol was pre-registered before data extraction. The analytic workflow described hereafter—eligibility criteria, search strategy, screening logic, and extraction schema—was defined a priori and locked before the final registry queries were executed, and is reported here in full to support reproducibility.

### 2.2. Eligibility Criteria

We targeted adult MDD with treatment resistance. Inclusion required an interventional study (Early Phase I to Phase IV; randomized or non-randomized; blinded or open-label) evaluating a therapeutic strategy intended to modify depressive symptomatology, functioning, or relapse/recurrence risk. We excluded purely observational designs, pediatric/adolescent populations, and studies primarily enrolling bipolar, psychotic, or schizophrenia spectrum disorders. Trials centered on ketamine, esketamine, or S-ketamine were pre-specified for exclusion at the cleaning stage. This decision rested on three converging considerations. First, ketamine and esketamine constitute a therapeutic stream with its own dedicated pipeline-mapping literature, including recent comprehensive syntheses [2,3], whose inclusion within the present perimeter would have been duplicative rather than complementary. Second, intranasal esketamine is no longer an investigational candidate but a regulatory-approved option for TRD in both the United States (FDA, 2019) and the European Union; its position in the therapeutic landscape is thus structurally different from that of the developmental programs the present work set out to characterize. Third, given the disproportionate representation of (es)ketamine programs in current registries, their inclusion would have saturated the analytic signal and obscured the comparatively smaller—but conceptually distinctive—strata of device-based, digital–hybrid, and biologic/novel interventions that motivate this mapping. To minimize false negatives at retrieval, ketamine-class agents were allowed at the search stage and subsequently removed by case-insensitive matching across titles, intervention fields, and brief descriptions, including brand names and common variants (e.g., Spravato, Ketalar, Ketanest). Perinatal/postpartum depression and other special populations were considered out of scope unless explicitly labeled as TRD within the registry record.

### 2.3. Study Selection, Deduplication, and Audit Trail

Since many trials are multi-registered, we performed cross-registry deduplication using a deterministic hierarchy: ClinicalTrials.gov identifier (NCT) took precedence when present; otherwise, we used the CTIS/EU trial number, followed by ISRCTN. When none of these were available, a temporary placeholder based on the hashed scientific/public title was generated to avoid record loss pending manual verification. Secondary keys (sponsor/protocol number, country/site lists, and intervention labels) were used to consolidate near duplicates. All transformations were logged; master tables, intermediate exclusion lists, and versioned outputs were time stamped and archived to preserve an audit trail from raw exports to the locked analytic dataset.

### 2.4. Operationalization of TRD Within Registries

Recognizing that registry entries variably encode resistance, we implemented a tiered flag: explicit TRD when the record included canonical wording (e.g., “treatment-resistant depression,” TRD, “refractory/resistant depression”) or staging frameworks (e.g., Thase & Rush, Maudsley) or an explicit threshold indicating ≥2 adequate failures; implicit TRD when resistance was inferred from phrases such as “inadequate/partial/non response” or “non remission” without an explicit TRD label; and absent when no resistance language was detected. Ambiguous cases were pooled for manual adjudication prior to lock; in four instances judged clinically on target (augmentation with N acetylcysteine in refractory MDD; L leucine crossover in MDD; minocycline in MDD with CRP defined low-grade inflammation; D cycloserine augmentation of iTBS), we retained the records and labeled them accepted_absent to make the exception explicit.

### 2.5. Data Extraction and Variable Schema

For each unique trial, we extracted registry identifiers (NCT, EU/CTIS, ISRCTN), country and sponsor information, study phase, status, enrollment, population descriptors (including the verbatim TRD definition when present), intervention(s), comparator(s), and outcome measures. Data charting was performed by a single reviewer (F.M.) using a pre-specified structured schema comprising thirty-seven variables per trial, including registry identifiers, population descriptors, intervention and comparator text, phase, status, sponsor, primary and secondary endpoints, and two derived classification fields—the intervention-class assignment with a confidence tag (high/moderate/low) and the TRD flag (explicit vs. accepted_absent)—each paired with a rationale field recording the exact registry text or rule-match that justified the coding decision. No independent double-extraction was performed; the charting schema and its decision trail—preserved across the sequence of pipeline artefacts (raw registry exports, merged master, cleaned master, excluded records, dropped-after-review records, and final analytic dataset with endpoint flags)—are available on reasonable request to enable external audit.

Intervention-class assignment proceeded in two passes. In a first pass, a rule-based classifier applied broad keyword pattern recognition to titles, intervention fields, and brief descriptions to assign trials to four preliminary mechanism families (pharmacological, device-based, biologic/novel, and a heterogeneous digital/combined bucket aggregating digital therapeutics and hybrid protocols). The output of this first pass was then refined through a second-pass classifier anchored on (i) the structured intervention-tag types recorded in the source registries (e.g., DRUG, DEVICE, BEHAVIORAL, BIOLOGICAL, DIETARY_SUPPLEMENT, COMBINATION_PRODUCT) as the primary signal of intervention modality, and (ii) whole-word regular-expression matching across the trial title and the intervention text—but not the free-text description, since descriptive passages in depression trials routinely reference the broader therapeutic landscape (e.g., antidepressants, ECT, light therapy) in ways that do not reflect the intervention under investigation. Two filters were applied to reduce false positives: DEVICE-tagged interventions whose name corresponded to diagnostic-only modalities (e.g., MRI, fMRI, EEG, PET, polysomnography) were excluded from the device signal; and BEHAVIORAL-tagged interventions whose name was a research artefact (e.g., research interview, questionnaire, brain scan, fMRI task) rather than a therapeutic component were excluded from the psychotherapy signal. Generic descriptive terms commonly present in depression trials regardless of intervention type (“antidepressant,” “placebo”, “augmentation”) were not treated as drug signals on their own; the drug signal required either a structured DRUG tag or an explicit drug name.

The taxonomy that emerged from this two-pass classification comprised seven categories: device-based neuromodulation (rTMS, iTBS/TBS, deep TMS, tDCS, tACS, tRNS, ECT, MST, DBS, VNS/tVNS, photobiomodulation, neurofeedback, CES, focused ultrasound, epidural or prefrontal cortical stimulation, trigeminal nerve stimulation, PEMF, LFMS); pharmacological interventions (small molecules and pharmacogenomically guided drug-selection protocols, distinct from ketamine/esketamine which were excluded by design); biologic/novel agents (psychedelic-assisted protocols, monoclonal-antibody and cytokine-modulating strategies, fecal microbiota transplant, botulinum toxin, specialized pro-resolving mediators); multimodal non-digital combinations (device-plus-drug, device-plus-psychotherapy, drug-plus-psychotherapy, or drug-plus-lifestyle, without a digital component); digital–hybrid programs (digital therapeutics, virtual or augmented reality, telehealth-delivered therapy, wearable-based interventions, computerized cognitive training, avatar therapy, or any protocol incorporating a software-driven component); psychotherapy (manualized psychotherapy as the principal intervention, independent of device or pharmacological adjuncts); and lifestyle interventions (structured exercise, nutritional supplementation, sleep-deprivation or chronotherapy protocols, bright light therapy). A single trial whose principal intervention was a PET radiotracer for imaging of antidepressant pharmacology (NCT04841798) was assigned to the pharmacological category given that the substance under investigation was a pharmacological agent. The full decision rules, the per-trial assignment, and the audit fields (intervention tag, signal pattern, classification reason) are reported in the Supplementary Information (Supplementary Table S1).

### 2.6. Endpoint Harmonization

Given the variability of primary endpoints across the pipeline, we flagged a binary variable (endpoint_standard) as yes when the primary outcome explicitly referenced the Montgomery–Åsberg Depression Rating Scale (MADRS) or Hamilton Depression Rating Scale (HDRS/HAMD, including common variants) and as no otherwise; a companion field (endpoint_scale) recorded which scale was detected (MADRS, HDRS/HAMD, both, or none).

### 2.7. Sensitivity Safeguards and Exclusions

To reduce spurious exclusions, bipolar/psychosis and pediatric filters were applied to titles and condition labels rather than free text descriptions, thereby avoiding the removal of MDD TRD studies that merely listed these categories as exclusion criteria. Finally, where registry completeness was limited (notably in CTIS for narrative summaries), decisions defaulted to inclusion with manual tagging unless an exclusion criterion was unequivocally met.

### 2.8. Analytic Frame

This work is descriptive and cartographic; therefore, we did not conduct quantitative synthesis, risk-of-bias assessment, or comparative effectiveness analyses. Counts and distributions arising from the application of the above procedures will be reported in the results; here we limit ourselves to describing the pre-specified pipeline structure, the handling of definitional ambiguities in TRD, the mechanism-based taxonomy, and the endpoint flagging strategy that structures the reading of the registries.

## 3. Results

The registry interrogation yielded 400 unique interventional records before cleaning and de-duplication. After applying the pre-specified filters (adult MDD; interventional designs only; removal of ketamine/esketamine including stereochemical/brand variants; exclusion of bipolar/psychosis spectrum and pediatric populations) and consolidating cross-registry duplicates, 268 trials remained for tagging. A subset of 35 records without explicit resistance language (trd_flag = absent) was set aside for manual adjudication; 4 of these were retained as clinically concordant with TRD despite the absence of a verbatim label. The final analytic dataset comprised 237 trials, locked on 18 September 2025, and used for all descriptive summaries below.

When organized according to the analytic taxonomy, the pipeline was dominated by trials of device-based neuromodulation (114/237, 48.1%) and pharmacological strategies distinct from ketamine/esketamine (86/237, 36.3%), followed by biologic/novel agents (16/237, 6.8%), multimodal non-digital combinations (11/237, 4.6%), digital–hybrid programs (5/237, 2.1%), lifestyle interventions (3/237, 1.3%), and dedicated psychotherapy trials (2/237, 0.8%). Within the device class, the most frequent modalities were repetitive transcranial magnetic stimulation (rTMS) and intermittent theta-burst stimulation (iTBS), followed by transcranial direct current stimulation (tDCS), deep brain stimulation (DBS), electroconvulsive therapy (ECT), and vagus nerve stimulation (VNS). The biologic/novel stratum was populated mainly by psychedelics (psilocybin and 5-MeO-DMT compounds) and immunomodulatory agents (anti-TNF monoclonal antibodies, cytokine antagonists). With respect to resistance operationalization, records explicitly referencing treatment-resistant or refractory depression, staging frameworks, or explicit thresholds of multiple adequate failures comprised 233/237 (98.3%); 4/237 (1.7%) were retained as accepted_absent after manual review (augmentation with N-acetylcysteine in refractory MDD; L-leucine crossover; minocycline in MDD with CRP-defined low-grade inflammation; D-cycloserine augmentation of intermittent theta-burst stimulation). Primary endpoints were heterogeneous but skewed toward conventional depression rating scales. Trials with a standard clinical primary endpoint—a priori defined as MADRS or HDRS/HAMD (common variants included)—constituted 150/237 (63.3%), whereas 87/237 (36.7%) employed non-standard or surrogate primaries. Within the standard group, MADRS was identified in 75/237 (31.6%), HDRS/HAMD in 70/237 (29.5%), and both scales in 5/237 (2.1%); in the remainder, no such scale appeared in the primary outcome field (Table 1).

Illustrative exemplars, offered strictly as non-interpretative signposts, include a single-modality device trial (transcranial direct current stimulation in TRD, NCT00667680; coded device), a pharmacological augmentation pathway (NCT00296686, sequential tranylcypromine with dextroamphetamine and triiodothyronine; coded pharmacological), a biologic/novel entry (NCT05800860, GH001; coded biologic-novel), a device-plus-drug combination without digital component (D-cycloserine augmentation of intermittent theta-burst stimulation, NCT05591677; coded multimodal non-digital), and a true digital–hybrid program (NCT07112261, Avatar Therapy in Virtual Reality for TRD; coded digital–hybrid). A detailed, machine-readable breakdown by intervention class, endpoint standardization, and resistance flag is summarized in Table 1. Phase information was variably reported across registries and often missing (not specified in 121 of 237, 51.1%). Among declared phases, Phase 2 predominated (48 of 237, 20.3%), followed by Phase 4 (26, 11.0%), Phase 3 (15, 6.3%), Phase 1 (12, 5.1%), Phase 1/2 (9, 3.8%), and Early Phase 1 or Phase 2/3 (each 3, 1.3%). Recruitment status at the snapshot was completed in 111 of 237 (46.8%), ongoing in 71 (30.0%), terminated/withdrawn in 28 (11.8%), and unknown in 27 (11.4%). Reported sample sizes (available for 236 records) spanned a wide range (median 49 [IQR 19–87], range 0–976), highlighting the predominance of small-to-medium studies (see Table 2).

Expanded geographic, temporal, and sponsorship analyses are reported in the Supplementary Information (see Supplementary Results).

Taken together, these descriptive findings delineate a pipeline that is both active and diversified, with device-based neuromodulation and pharmacology with novel mechanisms jointly accounting for the large majority of the active landscape, biologic/novel agents and multimodal combinations occupying smaller but distinctive strata, and purely digital–hybrid programs still marginal at the cut-off date. The trial size profile—while sufficient for signal detection—remains limited for confirmatory inference. The subsequent sections will examine whether this configuration maps onto meaningful progress toward stratified, biomarker-informed care, or primarily reflects a multiplication of options within the constraints of current infrastructure.

## 4. Discussion

Surveying ClinicalTrials.gov, EU CTIS and ISRCTN at the 18 September 2025 cut-off under our analytic eligibility and taxonomy, the development pipeline for MDD-TRD is organized around two co-active developmental tracks of comparable weight—device-based neuromodulation and pharmacological strategies distinct from ketamine/esketamine—with biologic/novel agents emerging as a small but distinctive third stratum and a residual minority of multimodal combinations, digital–hybrid programs, lifestyle interventions, and dedicated psychotherapy trials. In the consolidated dataset, device-based neuromodulation accounts for 114/237 (48.1%), pharmacological strategies for 86/237 (36.3%), biologic/novel agents for 16/237 (6.8%), multimodal non-digital combinations for 11/237 (4.6%), digital–hybrid programs for 5/237 (2.1%), lifestyle interventions for 3/237 (1.3%), and psychotherapy for 2/237 (0.8%) (Table 1 and Table 2). This profile is consistent with a clinical landscape in which accelerated iTBS has become an operational alternative to 10 Hz rTMS on clinician-rated outcomes while shortening session time [5,6,7], in which a new generation of pharmacological candidates—anti-inflammatory and glutamatergic agents, anaesthetic adjuncts, neurosteroid derivatives—is being tested under TRD eligibility frameworks alongside long-established augmentation strategies, and in which psychedelic-assisted protocols and immunomodulatory monoclonal antibodies are moving from proof of concept toward larger Phase 2 designs.

Developmental staging is incompletely specified in the registries (a known feature for pragmatic and academic studies): “phase not specified” appears in 121/237 (51.1%) entries. Among explicit designations, Phase 2 predominates (48/237; 20.3%), with Phase 4 (26/237; 11.0%) and Phase 3 (15/237; 6.3%) following (Table 2). The Class × Phase panel reflects a practical division of labor that is now visible across modalities: pharmacological programs concentrate in Phase 2 testing (36/86 entries, 41.9%) consistent with new-mechanism evaluation, while biologic/novel agents—psychedelics and immunomodulators most prominently—show an even stronger Phase 2 skew (9/16, 56.2%) compatible with current proof-of-concept programs; device-based entries are overwhelmingly recorded with no phase information (96/114, 84.2%), an idiosyncrasy of registry conventions for non-pharmacological interventions rather than a substantive absence of staging; and multimodal non-digital combinations show a Phase 4 skew (3/11, 27.3%) consistent with adjunctive and maintenance contexts. Outcome measurement is only partly standardized. Our endpoint_standard flag is met in 150/237 (63.3%) studies—i.e., a MADRS or HAMD/HDRS primary endpoint—with class-level gradients (≈64.0% in device-based; ≈59.3% in pharmacological; ≈62.5% in biologic/novel; ≈81.8% in multimodal non-digital) (Table 1). This heterogeneity matters for cross-trial comparability and evidence cumulation in neuromodulation, where recent syntheses report broadly convergent effects across iTBS and 10 Hz rTMS when measured on shared clinician-rated scales and where randomized tDCS + rTMS combinations have been tested explicitly under HAMD-24 primaries [6,7,8]. Sample sizes underline an exploratory posture: the median planned enrollment is 49 [IQR 19–87], with larger medians in biologic/novel and multimodal non-digital entries (~70 each) and smaller medians in single-modality device (42) and pharmacological (44) trials (Table 2). In parallel, anchor points in contemporary evidence help interpret the map without importing them into our counts: ECT remains the historical benchmark whose cognitive trade-offs can be mitigated by modern dosing and lead placement [9], while a pragmatic non-inferiority trial in nonpsychotic TRD positioned IV ketamine against ECT for short-term response—background that is relevant even though (es)ketamine is excluded by design from our dataset [10]. Digital programs, on the other hand, draw on the literature in which therapist-guided iCBT outperforms unguided formats at higher baseline severity and in which face-to-face advantages attenuate when guidance, duration and adherence are handled—features that contextualize the small but distinctive digital–hybrid stratum we observe in the registries [11,12]. Current articulates this division of labor by recommending rTMS/iTBS after antidepressant non-response within measurement-based care, while maintaining ECT as a priority in urgent or psychotic presentations [13].

### 4.1. Non-Convulsive Neuromodulation in Context (rTMS/iTBS, tES, and Combinations)

The registry pattern that concentrates device-based entries in mid-development phases dovetails with a body of evidence in which intermittent theta burst stimulation (iTBS) has been positioned as a time-efficient alternative to conventional 10 Hz rTMS on clinician-rated depressive outcomes, with non-inferiority demonstrated in a large multicentre trial and consolidated across comparative syntheses and practice statements [5,6,7,14]. Under safety parameters codified by international consensus, seizure risk remains very low and adverse effects are typically transient (headache, scalp discomfort), a profile that has supported service-level adoption and optimization work rather than de novo efficacy testing [15,16]. In parallel, accelerated schedules have re-oriented clinical questions from if to how fast meaningful change can be detected: neuroscience-informed accelerated iTBS protocols (SAINT/SNT) have yielded rapid improvements under sham controlled conditions, while reviews caution that dosing intensity, inter-session spacing and targeting procedures complicate generalization to routine pathways [17,18]. A second arc in the registries—the testing of transcranial electrical stimulation (tES) alone or grafted onto magnetic protocols—tracks a literature that has moved beyond single-centre tDCS signals to broader syntheses and exploratory work on tACS. A recent JAMA Network Open meta-analysis spanning tDCS, tACS and tRNS reports overall antidepressant effects for tDCS (with heterogeneity across diagnostic strata), promising but still small sample signals for tACS, and limited evidence for tRNS; combinations with medication appear favorable but require larger confirmatory trials [19]. In this methodological landscape, factorial device–device strategies are an empiric route to earlier symptom change under familiar primary measures: a multicentre four arm RCT found larger HAMD 24 improvement at two weeks for tDCS + rTMS than either monotherapy with good tolerability, a design logic echoed by the hybrid and adjunctive entries in our registry map [8]. In our registry sample, the prominence of hybrid and adjunctive designs in neighboring families—multimodal non-digital combinations and the smaller digital–hybrid stratum—is consistent with this direction of travel, in which neuromodulation is less a stand-alone alternative and more a platform onto which pharmacological, behavioral and sometimes digital elements are grafted, shifting the evidentiary questions from “if” to “how fast”, “for whom”, and “with what scheduling constraints”, questions that our Class × Phase distribution and endpoint choices make tractable without implying comparative conclusions [5,8]. While the speed of clinical response under accelerated dosing appears plausible, durability and maintenance of effect remain challenges that future schedules must deliberately construct rather than assume.

### 4.2. Convulsive Approaches in Contemporary TRD (ECT with a Brief Note on MST)

Convulsive therapies constitute a mature stratum where the methodological emphasis has shifted from demonstrating the existence of antidepressant effects—which are long documented—to refining how such effects can be delivered with preserved efficacy and attenuated cognitive burden. Programmatic syntheses converge on a small set of levers—stimulus dose, pulse width, and electrode placement—that shape this balance. In this sense, convulsive entries in the registries—typically anchored to MADRS or HAMD primaries in line with our endpoint_standard flag—track pragmatic questions of “how to deliver and to whom” rather than “whether ECT works,” reflecting a mature intervention re-examined through cognitive parameters as much as through symptomatic endpoints. Comparative context remains relevant even though it lies outside our registry corpus by design. In non-psychotic TRD, a pragmatic randomized non-inferiority trial found intravenous ketamine non-inferior to ECT for short-term response, with the expected divergence in adverse-event profiles and early memory effects [10]. While (es)ketamine is excluded from our dataset, this signal underscores why convulsive therapies continue to serve as the benchmark against which somatic and hybrid programs are implicitly calibrated, particularly in scenarios requiring rapid and robust symptom reduction. Current guidance maintains this stratification: ECT is prioritized when urgency, psychosis, catatonia, or severe functional compromise dictate decision-making, whereas rTMS and iTBS are positioned earlier in measurement-based care after antidepressant nonresponse, a division of labor visible in our Class × Phase distributions [13]. In parallel, magnetic seizure therapy (MST) has emerged as an experimental attempt to induce the therapeutic seizure with more focal magnetic fields. Systematic reviews suggest antidepressant efficacy broadly comparable to ECT with shorter recovery and reorientation times and smaller decrements in immediate and delayed recall and verbal fluency, although extant samples remain small and heterogeneous and long-term cognitive trajectories await phase-advancing confirmation [20,21].

### 4.3. Biologics/Novel Beyond Ketamine: What Psilocybin May Contribute and What It Might Not

The biologics/novel slice of the registry map is small in absolute terms yet informative for how symptom change is being operationalized Psilocybin entries sit almost uniformly in early phase development and, in keeping with our endpoint_standard variable, anchor efficacy to clinician-rated depression severity on the MADRS rather than mixed composites—a choice that aligns the field’s principal dose finding and durability questions with the scales used in device and pharmacological comparators. The pivotal phase 2b randomized trial in treatment-resistant depression administered a single dose of 25 mg, 10 mg, or 1 mg synthetic psilocybin with structured psychological support and specified the MADRS change at week 3 as the primary endpoint; least squares mean change favored 25 mg over 1 mg (−12.0 vs. −5.4; difference −6.6 [95% CI, −10.2 to −2.9]), with separation detectable as early as day 2, while 10 mg did not differ from 1 mg at the primary time point [22]. Adverse events were frequent but mostly transient (headache, nausea, dizziness), and suicidal ideation/behavior/self-injury occurred in all dose groups, underscoring the need for systematic monitoring within the supportive framework that is intrinsic to current protocols [22]. Methodologically, these designs must accommodate functional unblinding and expectancy—inevitable with macrodose psychedelics—in ways that preserve interpretability; contemporary analyses and commentaries highlight blinding integrity as a central threat to internal validity in this domain [23,24,25,26]. The 52-week COMP004 observational follow-up enrolled a subset of participants after completion of the phase 2b trial and examined time to first depressive event (a composite including initiation of new treatment, hospitalization, suicidality, or pre-specified MADRS worsening); the median time to event was longer after 25 mg than after 1 mg, with descriptive patterns consistent with more durable benefit at the higher dose, though the study was unpowered, subject to selection bias, and permitted naturalistic co-interventions after week 12 [27]. Adjacent MDD trials help contextualize measurement choices and effect size geometry across diagnoses. A multicenter phase 2 RCT in MDD using MADRS and centralized blinded raters found that a single 25 mg session plus psychological support produced rapid and sustained reductions over six weeks versus niacin as active placebo, reinforcing that current efficacy claims are articulated primarily on clinician-rated scales [28]. By contrast, the escitalopram comparison in non-TRD MDD—two 25 mg psilocybin sessions three weeks apart versus daily SSRI—showed no between-group difference on the pre-specified QIDS SR 16 at six weeks, while several secondary outcomes favored psilocybin; longer-term follow-up has emphasized functional and psychosocial dimensions rather than primary symptom scores, illustrating how outcome selection influences interpretation [29,30]. Meta-analytic summaries through 2024–2025 converge on moderate to large short-term symptom reductions and suggest dose response advantages near 25 mg, while emphasizing heterogeneity, small samples, and durability uncertainties that phase advancing trials must resolve [31,32,33,34]. Methodological audits further note that control arm performance in psilocybin trials differs from that seen with SSRIs or esketamine in antidepressant programs, a pattern that interacts with blinding/expectancy and complicates cross-program comparisons—even when endpoint scales nominally align [35].

### 4.4. Digital and Hybrid Interventions

Although digital–hybrid programs in our registry mapping constitute a small stratum (5/237, 2.1%), the surrounding literature on internet-delivered CBT, app-based modules with coaching, and blended formats that integrate digital components into therapist-delivered care is substantial and informs how these trials should be read. Two robust summaries anchor what these programs achieve and under which conditions. First, an IPD network meta-analysis in depression showed that guided iCBT outperforms unguided iCBT on short-term PHQ-9, with moderation by baseline severity—advantages concentrate in the moderate–severe range, while unguided formats approximate guided care only at mild/subthreshold symptom levels [11]. Second, a comparative meta-analysis across 106 studies (*n* ≈ 11,854) reported larger pre–post effects and better adherence for face-to-face CBT, yet differences attenuated after propensity matching for baseline severity and other imbalances; the same analysis identified human support, longer duration and adherence as moderators of digital outcomes [12]. Equivalence claims have been revisited with contemporary data. A recent synthesis argues that therapist-guided iCBT can be clinically comparable to face-to-face CBT when delivered under guideline-concordant conditions, sharpening the interpretation of registry entries that position digital modules as substitutes or amplifiers rather than adjuncts of convenience [36]. The blended frontier is particularly relevant to our taxonomy: a 2024 meta-analysis of blended therapy indicates improved access and uptake with effects aligned to conventional psychotherapy, while an RCT in major depression found brief blended CBT (short in-person sessions plus smartphone content) clinically non-inferior to full face-to-face care in routine services [37,38]. These results resonate with the position of digital programs in the broader TRD landscape, where they more often serve adjunctive or maintenance roles than primary therapeutic substitutes, and may help explain why the registered digital–hybrid pipeline remains small relative to the maturity of the underlying evidence base. Engagement and attrition remain variable and context-dependent rather than uniformly poor; real-world patterns include a subset of “superusers” alongside early drop-off, with design, user and system factors jointly shaping retention [39,40]. Meta-research using multiverse approaches documents a “vibration of effects” for digital interventions in depression, with average Hedges g ≈ 0.43 across analytic specifications and smaller effects after bias adjustment or >24-week follow-up; effects are larger with guidance, adult samples and waitlist comparators [41]. Looking ahead, the strongest registry signals suggest a transition from digital tools as peripheral aids toward configurations where software functions as an intrinsic therapeutic mechanism—scaffolding adherence, structuring remote assessments, and extending clinical surveillance beyond the acute treatment window.

### 4.5. Endpoints and Outcome Measurement: MADRS vs. HAMD and Why Harmonization Matters

In our analytic set, 150 of 237 trials (63.3%) declared a clinician-rated depression scale as the primary endpoint, with class-level gradients (≈64.0% among device-based entries; ≈59.3% in pharmacological; ≈62.5% in biologic/novel; ≈81.8% in multimodal non-digital) and a residual third relying on non-standard or surrogate measures (Table 1). By construction, the two scales are not interchangeable: the HAMD-17 is broader and multidimensional, with heavier weighting of insomnia and somatic items, whereas the MADRS was explicitly designed to capture core mood and cognitive symptoms and has increasingly become the preferred endpoint in modern neuromodulation trials, even though many seminal rTMS studies used HAMD [42]. Large-scale comparative evidence suggests, however, that the average treatment effects do not diverge systematically. A recent meta-analysis pooling 109 HAMD-17 trials (*n* = 32,399) and 28 MADRS trials (*n* = 11,705) found near-identical standardized drug–placebo differences (SMD 0.27 vs. 0.30) and small raw gaps—≈2.07 points on HAMD-17 and ≈2.99 on MADRS—both below the thresholds commonly invoked as minimal clinically important differences (≈7–8 points, or 7–9% of scale maximum) [43]. An rTMS-specific crosswalk, built on the FOURD and CARTBIND randomized datasets (N = 380), compared five statistical models: linear regression (RMSE ≈ 2.66–4.82), support vector regression close behind, and an rTMS-equipercentile solution performing intermediately overall but with less systematic bias in Supplementary Analysis. The authors provide ready-to-use translation tables and caution against out-of-range conversions, as model performance deteriorates when baseline severity distributions deviate from calibration sets [42]. In our reading, such crosswalks are best understood as calibration aids for sensitivity analyses rather than wholesale replacements for pre-specified endpoints. Finally, none of these harmonization strategies succeed without measurement quality. Multicentre trials are susceptible to rater drift and inter-rater variability; structured training, web-based calibration, and ongoing reliability checks reduce noise and stabilize estimates, prerequisites for any meaningful cross-program synthesis [44,45]. Against this backdrop, our reliance on endpoint_standard ensures a common currency across classes, while our analytic stance acknowledges scale-specific geometry, uses validated HAMD-MADRS crosswalks where appropriate—particularly in rTMS/iTBS—and retains explicit reporting of native endpoints to preserve transparency around symptom domains and timing choices. A further dimension of endpoint heterogeneity, only partly addressed by the MADRS/HAMD harmonization debate, concerns the under-representation of cognitive and functional outcomes in the registered pipeline. Symptom-rating scales—even when consistently chosen across modalities—capture neither the cognitive sequelae that frequently accompany resistant illness (executive dysfunction, attentional impairment, memory complaints) nor the functional and psychosocial recovery that patients themselves identify as priority outcomes. In our analytic set, dedicated cognitive endpoints (e.g., MATRICS Consensus Cognitive Battery, BAC-A, or domain-specific neuropsychological batteries) appear infrequently as primary outcomes, and validated functional or work-related measures (WHODAS 2.0, Sheehan Disability Scale, FAST, WSAS) more often occupy secondary or exploratory positions than primary endpoint slots. This asymmetry is consequential: in domains such as ECT and accelerated iTBS, where cognitive trade-offs and time-to-functional-recovery are central clinical concerns, the symptom-rating frame may systematically under-detect both benefits and harms relevant to real-world decision-making. Future registered programs in TRD would benefit from co-primary or hierarchical outcome families that explicitly incorporate validated cognitive and functional measures alongside MADRS or HDRS/HAMD primaries, with follow-up windows aligned to the temporal horizon over which functional recovery is plausibly achieved.

### 4.6. Strengths and Limitations

Our approach is explicitly descriptive and follows the workflow formalized in the Methods Section—systematic interrogation of ClinicalTrials.gov, EU CTIS, and ISRCTN, deduplication across sources, intervention-class tagging through a two-pass classifier, and flagging of primary endpoints—yet several constraints merit transparency.

First, TRD is variably operationalized across registry entries; we therefore recorded the resistance criterion verbatim and applied a tiered flag distinguishing explicit declarations, implicit inferences, and accepted_absent cases adjudicated against the eligibility criteria. This choice preserves fidelity to the registry record and renders definitional heterogeneity visible at the trial level, but it does not—and cannot—retroactively harmonize what the underlying field still encodes inconsistently. The literature on TRD continues to span definitions ranging from failure of two adequate antidepressant trials to multidimensional staging frameworks incorporating episode duration, comorbidity, and patient-reported outcomes [1,2,3]; the European Group on Treatment-Resistant Depression has more recently proposed a consensus operationalization, but its uptake across registry entries remains partial. The 4/237 accepted_absent records we retained (1.7%) make the boundary of our inclusion explicit, while residual misclassification at study margins cannot be excluded, particularly for entries that infer resistance from broader phrasing without canonical TRD wording.

Second, data charting was performed by a single reviewer (F.M.) against a pre-specified structured schema with rationale fields recording the registry text or rule-match supporting each coding decision. We did not perform independent double-coding for inter-rater reliability, which is the principal methodological limitation of this work. We mitigated the associated risk by (i) anchoring intervention-class assignment on the structured intervention-tag types recorded in the source registries rather than on free-text descriptions, (ii) applying explicit filters to exclude diagnostic-only DEVICE tags and research-artefact BEHAVIORAL tags from therapeutic modality signals, and (iii) preserving an audit trail that allows external replication of the coding decisions from the published Supplementary Information. Nevertheless, single-reviewer charting remains susceptible to systematic bias that double coding would have detected, and future updates of this mapping should incorporate independent extraction.

Third, registry ecosystems are incomplete and overlapping: fields may be missing or inconsistently updated; cross-posting can generate near-duplicates despite deduplication rules; amendments and status changes lag, blurring recruitment and phase attribution. Fourth, a temporal cut-off (18 September 2025) anchors the map; programs initiated or updated thereafter are necessarily absent. Fifth, by design we excluded ketamine/esketamine, including branded and stereochemical variants, to maintain a coherent perimeter that complements the dedicated pipeline-mapping literature on these agents [2,3]; this improves internal comparability across classes while narrowing external contrasts with real-world care where these agents are available. Sixth, a non-trivial minority of trials declare nonstandard primaries (functioning, imaging, digital engagement), limiting synthesis even when class and phase are aligned; our endpoint_standard flag improves readability but cannot retroactively harmonize heterogeneous outcomes.

Set against these limitations, the registry lens offers distinctive strengths: near-real-time tracking of where investment flows and how designs mature; a taxonomy by intervention class that makes visible the relative weight of pharmacological, device-based, biologic/novel, and hybrid programs; and systematic visibility of endpoints and timing choices that rarely surface in early publications, enabling a method-first view of the pipeline rather than a post hoc narrative.

### 4.7. Towards Stratified, Biomarker-Informed Care: An Emerging Direction

Viewed through a registry perspective, the diversification of interventions points toward the need for trials that extend their scope beyond short-term symptom reduction and address questions of durability, functioning, and patient-reported outcomes. Follow-up windows longer than the customary 6–8 weeks, time-to-event metrics for relapse or retreatment, and co-primary or hierarchical families that combine clinician-rated scales with validated PROs would align outcome assessment with patient-relevant change. The pipeline we observe, however, does not yet pervasively reflect this shift: stratification by mechanism-proximal biomarkers and by circuit-level phenotypes remains an emerging research direction concentrated in specific strata—biologic/novel agents (where inflammation-informed enrolment is increasingly visible), a minority of device-based programs (where EEG and TMS-EEG signatures are being explored as allocation aids), and a small number of pharmacological entries explicitly anchored to genotype or transcriptomic readouts—rather than a pervasive feature of the current trial landscape. Subgroup analyses have shown that patients with elevated inflammatory markers, such as high CRP, may respond preferentially to immune modulators, with enrichment strategies yielding effects that are more reproducible than episodic [46]. In parallel, efforts to define EEG and TMS-EEG phenotypes and to build circuit-level stratification scores suggest possible pathways toward allocation and early futility decisions in stimulation programs [47,48,49]. These developments align with the growing application of adaptive platform designs, which permit shared controls, time-trend adjustment, and early retirement of non-promising arms while sustaining multi-arm learning [50,51]. At the point of care, the CANMAT 2023 update emphasizes stepwise, measurement-based, and personalized management. Within this framework, the integration of digital tools with somatic programs—for adherence monitoring, remote assessments, or combined therapeutic delivery—can be pursued without departing from established standards of evidence [13], even as the registry mapping suggests that such integration is more often discussed in the literature than instantiated in active trials at the present cut-off.

### 4.8. Clinical Gap and Unmet Need

**Figure 2:**
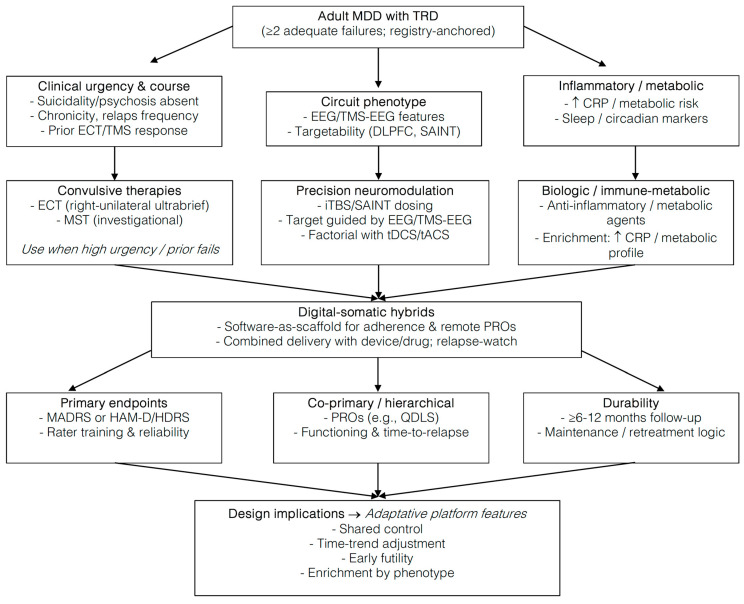
Conceptual stratified-care framework for treatment-resistant depression (TRD), integrating clinical urgency, circuit, and inflammatory phenotypes with digital-somatic hybrid design logic. Arrows indicate clinical decision pathways and the integration of therapeutic modalities.

A central practical implication of our registry-anchored map is the recognition that approximately one-third of patients with MDD do not achieve remission with conventional antidepressant sequences, even after multiple adequate trials. This figure, which has been consistently reproduced across epidemiological and clinical datasets, defines the clinical reality of treatment resistance and underscores the urgency of developing alternatives. Within this perspective, the distribution of programs observed in the registries—convulsive and non-convulsive neuromodulation, digital-somatic hybrids, and a small but expanding group of biologic and metabolic approaches—can be read not merely as a catalogue of ongoing experiments, but as the set of plausible avenues through which this unmet need may eventually be addressed. Each carries distinct promises and liabilities: neuromodulation offers rapid symptom change yet requires durable maintenance strategies; digital hybrids improve adherence and scalability but raise questions of engagement and data governance; biologic and metabolic trials are aligned with mechanistic enrichment but remain early-phase and underpowered. Taken together, these trajectories suggest that the therapeutic future of TRD will not be defined by a single breakthrough but by the deliberate integration of multiple modalities, evaluated against the benchmark that at least one-third of patients remain underserved by existing options. The registry map thus functions as a structured reminder that the success of the next generation of interventions will be judged not only by short-term efficacy but by their capacity to reduce the non-responder fraction in routine care. An overview of phenotype-driven allocation, endpoint structure, and adaptive design logic is provided in Figure 2.

Additional commentary and literature context on neuromodulation, biologics, digital–hybrid designs, and outcome measurement are presented in the Supplementary Information (see Supplementary Discussion).

## 5. Conclusions

The mapping presented herein, grounded in public registries and delimited by a transparent methodological perimeter, offers a composite picture of 237 ongoing or completed interventional programs targeting adults with treatment-resistant major depressive disorder. Far from aspiring to exhaustiveness—an aim no registry snapshot could credibly achieve—its value lies in the clarity it brings to a field often obscured by fragmentation, provided that its boundaries are acknowledged: heterogeneous definitions of treatment resistance, missing data fields, a fixed temporal cut-off, and the deliberate exclusion of ketamine and esketamine trials. Within these limits, the distribution of programs reveals a landscape in which device-based neuromodulation and pharmacology with novel mechanisms together account for the large majority of active development, while biologic/novel agents—most prominently psychedelics and immunomodulators—represent an expanding minority stratum, and convulsive therapies and digital–hybrid programs occupy distinct but smaller positions.

The comparability of findings across these modalities will hinge on the precision of measurement and on design choices appropriate to heterogeneity. Harmonization between MADRS and HAMD/HDRS, the elevation of patient-reported outcomes and durability measures to formal outcome families, and the use of adaptive platform structures with mechanism-proximal enrichment where warranted are the methodological premises on which credible cross-program synthesis depends. Considerations of equity, implementability, and data governance—elaborated in the Supplementary Information (see Supplementary Conclusions)—must be incorporated ex ante rather than retrofitted at the production stage.

Taken together, this registry-anchored snapshot does not propose a singular breakthrough, nor does it pretend to resolve the enduring intractability of treatment-resistant depression. Rather, it points to a near-term evolution of care founded on the deliberate integration of multiple therapeutic modalities, assessed through harmonized endpoints and designs sensitive to durability, and ultimately judged by their capacity to reduce the fraction of non-responders that current clinical practice continues to leave untouched.

## Figures and Tables

**Table 1:** Intervention classes × Standard clinical endpoint (MADRS/HDRS as primary).

Intervention Class	Yes (MADRS/HDRS as Primary)	No	Total	Yes (%)
Device-based neuromodulation	73	41	114	64.0
Pharmacological interventions †	51	35	86	59.3
Biologics/novel agents	10	6	16	62.5
Multimodal non-digital combinations	9	2	11	81.8
Digital–hybrid programs	3	2	5	60.0
Lifestyle interventions	2	1	3	66.7
Psychotherapy	2	0	2	100.0
Total (*n* = 237)	150	87	237	63.3

Distribution of trials by intervention class according to whether the primary endpoint explicitly used a standard clinician-rated depression scale. Percentages refer to the proportion of studies within each intervention class. † One trial originally not classifiable by mechanism (NCT04841798, an imaging study of MAO-B receptor occupancy by tranylcypromine) was folded into the pharmacological category, since the intervention under investigation is a pharmacological agent assessed through PET radiotracer methodology. Abbreviations: MADRS, Montgomery–Åsberg Depression Rating Scale; HAMD/HDRS, Hamilton Depression Rating Scale.

**Table 2:** Intervention classes × Phase (*n*, % within row).

Intervention Class	Early Phase 1	Phase 1	Phase 1/2	Phase 2	Phase 2/3	Phase 3	Phase 4	Not Specified	Total
Device-based neuromodulation	0 (0.0%)	3 (2.6%)	3 (2.6%)	1 (0.9%)	2 (1.8%)	5 (4.4%)	4 (3.5%)	96 (84.2%)	114 (100.0%)
Pharmacological interventions †	3 (3.5%)	8 (9.3%)	4 (4.7%)	36 (41.9%)	0 (0.0%)	10 (11.6%)	17 (19.8%)	8 (9.3%)	86 (100.0%)
Biologic/novel agents	0 (0.0%)	1 (6.2%)	2 (12.5%)	9 (56.2%)	1 (6.2%)	0 (0.0%)	1 (6.2%)	2 (12.5%)	16 (100.0%)
Multimodal non-digital combinations	0 (0.0%)	0 (0.0%)	0 (0.0%)	2 (18.2%)	0 (0.0%)	0 (0.0%)	3 (27.3%)	6 (54.5%)	11 (100.0%)
Digital–hybrid programs	0 (0.0%)	0 (0.0%)	0 (0.0%)	0 (0.0%)	0 (0.0%)	0 (0.0%)	0 (0.0%)	5 (100.0%)	5 (100.0%)
Lifestyle interventions	0 (0.0%)	0 (0.0%)	0 (0.0%)	0 (0.0%)	0 (0.0%)	0 (0.0%)	1 (33.3%)	2 (66.7%)	3 (100.0%)
Psychotherapy	0 (0.0%)	0 (0.0%)	0 (0.0%)	0 (0.0%)	0 (0.0%)	0 (0.0%)	0 (0.0%)	2 (100.0%)	2 (100.0%)
Total (*n* = 237)	3 (1.3%)	12 (5.1%)	9 (3.8%)	48 (20.3%)	3 (1.3%)	15 (6.3%)	26 (11.0%)	121 (51.1%)	237 (100.0%)

Reported trial phases by intervention class, as specified in registry entries across ClinicalTrials.gov, EU CTIS, and ISRCTN. Phase data are as entered in registries; “not specified” indicates missing or incomplete fields. Percentages refer to proportions within each intervention class. † See Table 1 footnote.

## Data Availability

All primary data analyzed in this study were obtained from three public clinical trial registries: ClinicalTrials.gov (https://clinicaltrials.gov, accessed on 18 September 2025), the EU Clinical Trials Information System (https://euclinicaltrials.eu, accessed on 18 September 2025), and ISRCTN (https://www.isrctn.com, accessed on 18 September 2025). The de-identified analytic dataset compiled and used for this study, together with the full extraction variables and endpoint flags, is provided as Dataset S1 in the Supplementary Materials.

## Supplementary Materials

The following supporting information can be downloaded at: https://www.mdpi.com/article/10.3390/brainsci16060612/s1, Supplementary Materials S1 (expanded Introduction, Methods, Results, Discussion and Conclusions, including geographic, temporal, and sponsorship analyses); Dataset S1: de-identified analytic dataset of all 237 included trials with extraction variables and endpoint flags; Supplementary Table S1: classification audit reporting the seven-category taxonomy assignment, intervention-tag signals, decision rules, and per-trial rationale fields; PRISMA-ScR checklist.
