# Supplementary material for: From Resistance to Redesign—The Emerging Logic of Hybrid Care in Treatment-Resistant Depression
Source: Brain Sci. 2026 Jun 4;16(6):612. doi: 10.3390/brainsci16060612 (PMC13297414; doi:10.3390/brainsci16060612)
Supplement: Supplementary file 1 [file brainsci-16-00612-s001.zip › Supplementary Materials S1_Clean Version.pdf]

brain sciences

MDPI

Supplementary Information

From Resistance to Redesign—The Emerging Logic of Hybrid Care in Treatment-Resistant Depression

S1. Supplementary Introduction

S1.1. Burden & QoL/Work impact

The humanistic and economic burden associated with TRD has been consistently documented across health systems. Indeed, in the United States, claims-based analyses estimate that among approximately 8.9 million adults treated for MDD annually, nearly 2.8 million (30.9%) meet criteria for TRD. The annual economic burden attributable to this subgroup approaches 43.8 billion US dollars, representing almost half of the overall costs of pharmacologically treated MDD. The contribution of TRD seems disproportionate across domains: over half of the direct health care expenditures, nearly half of unemployment-related costs, and one-third of productivity losses can be ascribed to this population [1]. Similar findings emerge in Europe, where cross-sectional data from over 52,000 respondents reveal markedly impaired health-related quality of life among individuals with TRD, with decrements of −18.1 points on the mental component summary and −5.4 on the physical component summary compared to general population norms. Work productivity is likewise severely compromised, with relative risks of 2.7 for absenteeism and 2.5 for overall activity impairment, while utilization of health care resources is dramatically elevated [2].

*S1.2. Current options & limitations (STAR*D, ECT/rTMS, esketamine)*

Current treatment strategies for MDD remain largely anchored to monoaminergic modulation, a paradigm that, while effective for many, fails to achieve remission in a substantial proportion of patients. Data from the STAR*D trial show a progressive decline in remission rates across sequential steps of pharmacotherapy: from 36.8% at the first trial, to 30.6%, 13.7%, and 13.0% at subsequent steps, respectively [3]. Beyond the second failed attempt, the likelihood of sustained remission decreases dramatically, reflecting the clinical trajectory of treatment-resistant depression (TRD). Moreover, each failure is not neutral: it compounds the risk of chronicity, relapse, suicidality, and impaired functioning [4]. Augmentation strategies with lithium or second-generation antipsychotics, as well as combination regimens such as olanzapine–fluoxetine, have demonstrated to provide incremental benefits but are often constrained by metabolic, neurological, or cardiovascular side effects [4]. Electroconvulsive therapy (ECT) remains the most effective somatic intervention for TRD, yet its acceptability is hindered by stigma and adverse cognitive effects, particularly anterograde and retrograde amnesia [4]. Repetitive transcranial magnetic stimulation (rTMS) and deep brain stimulation (DBS) offer non-pharmacological alternatives, however evidence is limited by small sample sizes, heterogeneity of protocols, and high attrition rates [4]. The approval of intranasal esketamine has been hailed as the first genuine innovation in antidepressant pharmacotherapy since the advent of SSRIs. Randomized controlled trials demonstrate significant advantages in response and remission rates compared to oral antidepressants alone [5], and long-term maintenance studies report a 51–70% reduction in relapse risk among responders [6]. Nevertheless, the efficacy of esketamine remains partial: remission rates are below 50%, durability is uncertain, and

treatment is limited by the need for co-administration with oral antidepressants, controlled clinical settings, and monitoring for dissociative and cardiovascular effects [6]. Furthermore, ketamine itself—despite robust off-label evidence of rapid antidepressant action—remains excluded from regulatory approval in TRD owing to its expired patent status and consequent lack of industry-driven phase 3 trials [4].

S1.3. Contextual notes (ketamine/esketamine; personalization/biomarkers)

The trajectory of ketamine and esketamine illustrates, perhaps more clearly than any other example, both the promise and the limits of the current therapeutic armamentarium, since their rapid antidepressant effects are consistently demonstrated even as response rates remain variable; maintenance strategies continue to be debated; and the need for structured clinical infrastructure and close monitoring constrains scalability and, in turn, access within health systems. While the prospect of personalization anchored in clinical features and emerging biomarkers offers a plausible path to improved outcomes, the predictive capacity of most markers remains modest and requires integration into multivariate models before it can meaningfully inform clinical decisions [6].

S2. Supplementary Methods

S2.1. Search syntax & exports

On ClinicalTrials.gov, we used Condition/Disease = "Major Depressive Disorder" and Other terms = "treatment resistant" OR TRD OR "refractory depression", restricting to Interventional (Clinical Trial) and adult populations where available in filters; on CTIS, which does not support complex Boolean syntax, we executed two complementary queries using the Medical condition field ("Major depressive disorder" and "Treatment resistant depression"), limiting to Interventional trials in the psychiatry therapeutic area and adult/older adult groups; on ISRCTN we performed full text searches for "treatment resistant depression" OR TRD OR "refractory depression" and filtered to interventional designs. No language restrictions were applied. We exported complete result sets (including brief/study descriptions and primary/secondary outcomes) as comma separated values for uniform processing.

S2.2. Intervention classification & tagging

Initial assignment used rule based pattern recognition over titles, intervention labels, and descriptions; trials with overlapping signals were conservatively categorized as digital/combined; a confidence tag (high/moderate/low) was added to reflect the redundancy and specificity of the textual cues, and ambiguous cases were reviewed.

S2.3. Endpoint timing & non-standard primaries

Timepoints were extracted verbatim where specified but not standardized across studies at this stage; scale agnostic or surrogate primary endpoints (e.g., biomarkers, neuroimaging parameters, fatigue/functioning composites) were retained and coded as non standard. This convention was designed to enable subsequent stratification without prejudging the comparative value of exploratory readouts.

S2.4. Ketamine removal dictionary & historical-use rule

For ketamine class agents, brand and stereochemical variants were included in the removal dictionary to improve recall; records were preserved if ketamine was mentioned only as a historical treatment failure in eligibility criteria rather than as an active intervention or comparator.

S3. Supplementary Results

S3.1. Geography, temporal trends, sponsorship

Geographically, the most frequently represented countries were the United States (n≈108), Canada (≈41), China (≈15), Germany (≈13), Australia (≈12), and France (≈11), with additional contributions from Spain, Denmark, Israel, the United Kingdom, Austria, Belgium, Turkey, Italy, and Brazil. Grouped by macro region (counts not mutually exclusive because multi country studies contribute to multiple tallies), trials most often referenced North America, followed by Europe, with Asia/Middle East, Oceania, and Latin America/Africa contributing smaller yet non negligible fractions. Along the temporal axis, start years clustered in the most recent period, with visible inflection in 2021–2025 (peaks in 2021 and 2024). Sponsorship classifications, derived heuristically from free text labels and therefore to be interpreted cautiously, suggested a predominance of academic/public initiatives (≈65%), comparatively fewer industry sponsored protocols (≈8%), and a residual other/unclear category (≈27%). This distribution, while sensitive to registry nomenclature, is consistent with the impression of a field where investigator initiated work—particularly in neuromodulatory and hybrid designs—continues to account for a large share of activity.

S4. Supplementary Discussion

S4.1. Non convulsive neuromodulation

Independent reviews converge on left DLPFC tDCS as the most consistently supported montage in depression, while tACS shows advantage over sham in pooled RCTs at the price of substantial between study heterogeneity and sparse dose finding [7,8].

S4.2. Convulsive approaches

Meta-analytic evidence delineates these trade-offs: high-dose right-unilateral placement achieves antidepressant effects comparable to moderate-dose bitemporal while conferring advantages in reorientation time and retrograde memory [9]; ultrabrief-pulse right-unilateral stimulation further reduces impairments across cognitive domains, albeit at the cost of a modest increase in session number and a small decrement in acute mood efficacy, inviting individualized prioritization of outcomes. Across time, objective cognitive deficits tend to peak within the first 72 hours and normalize over subsequent weeks, with some domains even showing post-course improvement, consistent with a trajectory in which early impairments are transient under modern protocols [10]. Practice frameworks have absorbed these gradients by codifying parameter sets—e.g., right-unilateral brief or ultrabrief dosing algorithms—while delineating contexts in which ECT retains primacy. In this way, MST is positioned as part of a wider arc of innovation that aims to decouple clinical benefit from cognitive cost while retaining the convulsive mechanism as the core therapeutic driver. (See also additional data in the attached file: TRD_Master_final_with_endpoints_2025-09-18.xlsx).

S4.3. Biologics/novel

In the antecedent phase 2b population, sustained response at three months was proportionally higher in the 25 mg group than in lower dose controls, again within the constraints of enrichment and attrition that complicate long range inference in psychedelic programs [11]. Together with earlier open label work in TRD showing large within person improvements maintained to 3–6 months after two sessions (10 mg and 25 mg) with psychological support, the observational arc suggests a dose–durability relation that now requires confirmation in adequately powered randomized maintenance designs [12,13].

S4.4. Digital & hybrid

Against this background, the large scale psychotherapy literature situates CBT's expected effects relative to controls and alternative formats [14], while comparative work in depression clarifies that face to face advantages can narrow when moderators and adherence are explicitly handled—a pattern consistent with our registry's emphasis on hybrid

scaffolds to boost adherence and maintain endpoint standard measures [15]. Finally, the measurement substrate matters: many digital trials—unlike a majority of entries in our registries—lean on self report scales, and the npj Digital Medicine review explicitly mixed self and observer rated measures while noting higher face to face adherence [15], which will intersects directly with endpoint comparability (MADRS vs HAMD) and the harmonization required for cross class reading.

S4.5. Endpoints & outcome measurement

Parallel issues emerge in clinical interpretability. Anchor-based work mapping MADRS totals onto CGI-S severity categories clarifies the severity bands and supports design-stage power calculations, but the minimal clinically important difference (MCID) remains context-dependent, varying with baseline severity, rater frame, and training [16-18]. Moreover, the choice of scale can shift observed drug–placebo separations and remission rates—typically smaller on HAMD-17, larger on HAMD-6 or core item sets—so endpoint selection is not merely technical but epistemic, affecting the narrative of efficacy across classes [19]. Because our dataset mixes MADRS and HAMD primaries across adjacent programs, conversions become practically necessary to read the map in a unified metric. Pharmacotherapy-derived equipercentile links exist, but they may mis-translate outcomes when applied to neuromodulation because symptom trajectories differ between drug and device trials.

S4.6. Towards stratified biomarker-informed care

Anchor-based work, for example with the Quality of Life in Depression Scale (QLDS), has provided meaningful-change thresholds that can be paired with clinician-rated measures to enhance interpretability [20, 21]. Finally, our previous review on botulinum toxin A illustrated how an apparently unconventional intervention can be followed through the stages of mechanistic plausibility, early-phase signals, and safety characterization. This "pipeline thinking" underscores how registry-anchored maps may not only describe the distribution of ongoing programs but also anticipate how stratified, biomarker-informed care might be structured in forthcoming phases of development.

S5. Supplementary Conclusions

Against these opportunities stand clinical risks that must be made explicit at design stage. For psychedelic assisted care, the spectrum spans diversion and dependence adjacent concerns (salient for MDMA and, in a different register, nitrous oxide, with nutritional monitoring for B12 when relevant) despite psilocybin's low addictive profile; acute events—anxiety reactions, dissociation, hypertensive or other cardiovascular responses—requiring escalation pathways; and longer range uncertainties, including the possibility, however uncommon, of persistent perceptual disturbance, neurotoxicity signals, or switch to mania/psychosis in susceptible individuals, which argue for follow up horizons of at least 12–24 months. Methodological fragilities—functional unblinding, set/setting effects, and therapist effects—remain central threats to interpretability and should be prospectively mitigated rather than repaired post hoc. For neuromodulation, equity of access, durability of benefit outside expert centers, cross site standardization, and the small but non zero risk of mania switch or seizure deserve symmetrical attention, as do drug–device interactions in polypharmacy, the particular vulnerability of older adults, pregnancy, and cardiocerebral comorbidity. Across modalities, suicidality safety protocols should be pre specified, trained, and audited, with escalation triggers that are technically and organizationally feasible in routine services. Outcome measurement will determine whether apparent progress can be compared across programs and cultures. Durability needs to be elevated from a narrative claim to a formal outcome family—time to relapse or retreatment, hierarchical testing that extends beyond six to eight weeks, and maintenance

designs built into pivotal programs—so that effect geometry includes not only speed but staying power. Function and patient reported outcomes should be co primary or hierarchically protected, anchored to minimal clinically important differences (for example, with QLDS or Sheehan Disability Scale anchors) to preserve clinical meaning [21]. Because clinician rated primaries will continue to mix MADRS and HAMD/HDRS, prespecified cross walks should be treated as calibration aids rather than substitutes, accompanied by rigorous rater training and ongoing reliability checks to minimize drift. Generalizability requires independent replication and explicit cross cultural validation, including linguistic and health system adaptation of PROs, so that claims of effectiveness are not confined to a handful of high resource jurisdictions. In this sense, the endpoint flagging and harmonization logic used in this work—identifying standard clinician rated primaries and mapping how they are deployed—offers a practical template for future trials to declare comparability ex ante. The data backbone that will sustain these trajectories raises its own governance obligations. Continuous streams from apps, wearables, and remote assessments increase the risk of re identification and secondary use; dynamic consent, data minimization, explicit retention/deletion schedules, and traceable data lineage should therefore be default, not luxuries. Algorithms that inform allocation or safety need periodic bias audits and transparent reporting, with preplanned recalibration when data drift is detected; cybersecurity provisions and fail safes must extend to automated suicidality flags and human in the loop escalation. Interoperability—preferably via HL7 FHIR profiles—should be required so that digital scaffolds can be integrated into existing records without creating new silos or vendor lock in, and patient feedback should be designed to avoid information harm. Ethical and organizational considerations are inseparable from science: representative inclusion across gender, ethnicity, socioeconomic status and rurality; practical accessibility accounting for costs, caregiver time, and travel; recognition that therapist effects are real and therefore training and certification should be standardized; and sober, transparent public communication that neither hypes nor pathologizes, thereby protecting clinical practice from reputational boom and bust cycles. Methodologically, the most credible route forward might be to couple mechanism proximal enrichment with designs that learn efficiently and withstand scrutiny. Adaptive and platform trials with shared controls and time trend adjustment can accelerate development while limiting historical bias; preregistration with a statistical analysis plan and explicit stopping rules constrains flexibility at the points where expectancy, rater drift, and endpoint switching would otherwise converge. Real world evidence should be built on common data models with negative controls and target trial emulation to approximate causal contrasts, and post approval surveillance needs to be sized and tooled to detect rare but consequential events such as HPPD, neuropathies, or mania switch. The principal threats are visible in outline: the convergence of bias across expectancy, assessor drift, and flexible endpoints; privacy fragility and function creep; adverse selection in which effective technologies prove undeliverable at scale; recreational spillovers if substances are socially normalized without guardrails; and the clinical and economic sustainability of complex programs. Accordingly, plausible expectations for the next phase are not of universal cures but of better framed decisions: durability and PROs elevated to co primary status; robust enrichment along inflammatory and circuit level axes; deliberate integration of digital and somatic elements under explicit governance; endpoint harmonization planned before first patient in; and equity and implementability evaluated ex ante alongside efficacy. If pursued in this register, the field can move from proliferating options to stratified, biomarker informed care that is both methodologically credible and deliverable where patients actually live. Accordingly, the registry-based mapping offered here should not be read as present and/or future prescribing therapeutic pathways, but rather as delineating the questions that will inevitably shape the field. For clinicians, the central uncertainty concerns

whether signals observed across different modalities can translate into durable benefit once relapse-prevention and maintenance frameworks are systematically embedded. For researchers, the challenge is to clarify which enrichment approaches—whether inflammatory, metabolic, or circuit-based—can be applied in ways that improve stratification without narrowing external validity. For regulators and policy makers, the salient issues relate to equity of access, the stewardship of increasingly granular digital data streams, and the calibration of safety monitoring to risks such as suicidality, diversion, or function creep. Viewed in this light, the registry map underscores that durability, enrichment, equity, and governance are not optional refinements but structural nodes through which the next phase of development in treatment-resistant depression will necessarily pass.

S6. Supplementary References

1. Zhdanava M, Pilon D, Ghelerter I, Chow W, Joshi K, Lefebvre P, et al. The prevalence and national burden of treatment-resistant depression and major depressive disorder in the United States. J Clin Psychiatry 2021;82(2):20m13699. doi:10.4088/JCP.20m13699.
2. Jaffe DH, Rive B, Denee TR. The humanistic and economic burden of treatment-resistant depression in Europe: a cross-sectional study. BMC Psychiatry 2019;19:247. doi:10.1186/s12888-019-2222-4.
3. Rush AJ, Trivedi MH, Wisniewski SR, Nierenberg AA, Stewart JW, Warden D, et al. Acute and longer-term outcomes in depressed outpatients requiring one or several treatment steps: a STAR*D report. Am J Psychiatry 2006;163(11):1905–1917. doi:10.1176/ajp.2006.163.11.1905.
4. Brendle M, Ragnhildstveit A, Slayton M, Smart L, Cunningham S, Zimmerman MH, et al. Registered clinical trials investigating ketamine and esketamine for treatment-resistant depression: a systematic review. J Psychedelic Stud 2023;6(3):176–187. doi:10.1556/2054.2022.00234.
5. Papakostas GI, Salloum NC, Hock RS, Jha MK, Murrough JW, Mathew SJ, et al. Efficacy of esketamine augmentation in major depressive disorder: a meta-analysis. J Clin Psychiatry 2020;81(4):19r12889. doi:10.4088/JCP.19r12889.
6. Daly EJ, Trivedi MH, Janik A, Li H, Zhang Y, Li X, et al. Efficacy of esketamine nasal spray plus oral antidepressant treatment for relapse prevention in patients with treatment-resistant depression: a randomized clinical trial. JAMA Psychiatry 2019;76(9):893–903. doi:10.1001/jamapsychiatry.2019.1189.
7. Medeiros GC, Demo I, Goes FS, Zarate CA Jr, Gould TD. Personalized use of ketamine and esketamine for treatment-resistant depression. Transl Psychiatry 2024;14:481. doi:10.1038/s41398-024-03180-8.
8. Zheng EZ, Wong NML, Yang ASY, Lee TMC. Evaluating the effects of transcranial direct current stimulation on depressive and anxiety symptoms across conditions: an updated systematic review and meta-analysis. Transl Psychiatry 2024;14:295. doi:10.1038/s41398-024-03154-w.
9. Zheng W, Cai DB, Zhang QE, Nie S, Chen J, Huang XB, et al. Adjunctive transcranial alternating current stimulation for depression: a meta-analysis of randomized controlled trials. Front Psychiatry 2023;14:1154354. doi:10.3389/fpsyt.2023.1154354.
10. Kolshus E, Jelovac A, McLoughlin DM. Bitemporal v. high-dose right unilateral ECT for depression: systematic review and meta-analysis of randomized controlled trials. Psychol Med 2017;47:518–530. doi:10.1017/S0033291716002737.
11. Semkovska M, McLoughlin DM. Objective cognitive performance associated with electroconvulsive therapy for depression: a systematic review and meta-analysis. Biol Psychiatry 2010;68(6):568–577. doi:10.1016/j.biopsych.2010.06.009.
12. Goodwin GM, Aaronson ST, Alvarez O, Arden PC, Baker A, Bennett JC, et al. Single-dose psilocybin for a treatment-resistant episode of major depression: a phase 2b randomized trial. N Engl J Med 2022; 387: 1637–1648. doi:10.1056/NEJMoa2206443.
13. Carhart-Harris RL, Bolstridge M, Rucker J, Day CMJ, Watts R, Erritzoe DE, et al. Psilocybin with psychological support for treatment-resistant depression: an open-label feasibility study. Lancet Psychiatry 2016;3:619–627. doi:10.1016/S2215-0366(16)30065-7.
14. Carhart-Harris RL, Bolstridge M, Day CMJ, Rucker J, Watts R, Erritzoe DE, et al. Psilocybin with psychological support for treatment-resistant depression: six-month follow-up. Psychopharmacology (Berl) 2018;235(2):399–408. doi:10.1007/s00213-017-4771-x.
15. Cuijpers P, Miguel C, Harrer M, Plessen CY, Ciharova M, Ebert DD, et al. Cognitive behavior therapy vs control conditions, other psychotherapies, pharmacotherapies and combined treatment for depression: a comprehensive meta-analysis including 409 trials. World Psychiatry 2023;22:105–115. doi:10.1002/wps.21069.

16. Kambeitz-Ilankovic L, Rzayeva U, Völkel L, Wenzel J, Weiske J, Jessen F, et al. A systematic review of digital and face-to-face cognitive behavioral therapy for depression. npj Digit Med 2022;5:144. doi:10.1038/s41746-022-00677-8.
17. Masson SC, Tejani AM. Minimum clinically important differences identified for commonly used depression rating scales. J Clin Epidemiol 2013;66(7):805–807. doi:10.1016/j.jclinepi.2013.01.010.
18. Thase ME, Youakim JM, Skuban A, Hobart M, Zhang P, Augustine C, Montgomery SA. Evaluation of MADRS total score thresholds as indicators of clinical remission in patients with major depressive disorder and bipolar depression. J Affect Disord 2021;295:1178–1188. doi:10.1016/j.jad.2021.08.109.
19. Hori H, Yoshida T, Kunugi H, Inoue T. Assessment of minimum clinically important difference in depression: scoping review and implications. Front Psychiatry 2025;16:1556470. doi:10.3389/fpsyt.2025.1556470.
20. Lisinski A, Hieronymus F, Nilsson S, Eriksson E. Impact of chosen cutoff on response rate differences between selective serotonin reuptake inhibitors and placebo. Transl Psychiatry 2022;12(1):160. doi:10.1038/s41398-022-01882-5.
21. Rozjabek H, Li N, Hartmann H, Fu DJ, Canuso C, Jamieson C. Assessing the meaningful change threshold of Quality of Life in Depression Scale using data from two phase 3 studies of esketamine nasal spray. J Patient Rep Outcomes 2022;6(1):74. doi:10.1186/s41687-022-00453-y.
